# How to choose the right statistical test?

**DOI:** 10.4103/0301-4738.77005

**Published:** 2011

**Authors:** Barun K Nayak, Avijit Hazra

**Affiliations:** P. D. Hinduja National Hospital, Veer Savarkar Marg, Mumbai - 400 016, India; 1 Department of Pharmacololgy, Institute of Postgraduate Medical Education & Research (IPGME&;R), 244B Acharya J. C. Bose Road, Kolkata - 700020, India E-mail: ijo.editor@gmail.com

Today statistics provides the basis for inference in most medical research. Yet, for want of exposure to statistical theory and practice, it continues to be regarded as the Achilles heel by all concerned in the loop of research and publication – the researchers (authors), reviewers, editors and readers.

Most of us are familiar to some degree with descriptive statistical measures such as those of central tendency and those of dispersion. However, we falter at inferential statistics. This need not be the case, particularly with the widespread availability of powerful and at the same time user-friendly statistical software. As we have outlined below, a few fundamental considerations will lead one to select the appropriate statistical test for hypothesis testing. However, it is important that the appropriate statistical analysis is decided before starting the study, at the stage of planning itself, and the sample size chosen is optimum. These cannot be decided arbitrarily after the study is over and data have already been collected.

The great majority of studies can be tackled through a basket of some 30 tests from over a 100 that are in use. The test to be used depends upon the type of the research question being asked. The other determining factors are the type of data being analyzed and the number of groups or data sets involved in the study. The following schemes, based on five generic research questions, should help.[1]

**Figure 1:**
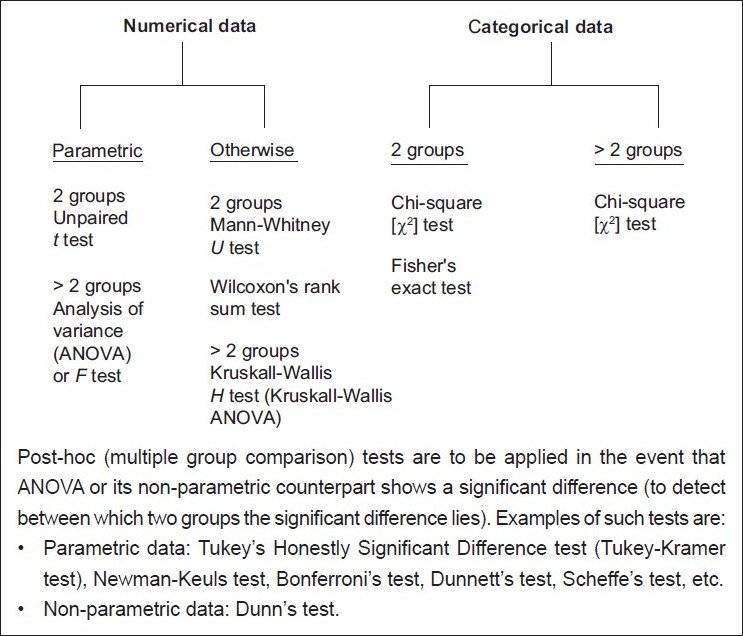
Tests to address the question: Is there a difference between groups – unpaired (parallel and independent groups) situation?

 **Question 1:** Is there a difference between groups that are unpaired? Groups or data sets are regarded as unpaired if there is no possibility of the values in one data set being related to or being influenced by the values in the other data sets. Different tests are required for quantitative or numerical data and qualitative or categorical data as shown in Fig. 1. For numerical data, it is important to decide if they follow the parameters of the normal distribution curve (Gaussian curve), in which case parametric tests are applied. If distribution of the data is not normal or if one is not sure about the distribution, it is safer to use non-parametric tests. When comparing more than two sets of numerical data, a multiple group comparison test such as one-way analysis of variance (ANOVA) or Kruskal-Wallis test should be used first. If they return a statistically significant *p* value (usually meaning *p* < 0.05) then only they should be followed by a post hoc test to determine between exactly which two data sets the difference lies. Repeatedly applying the t test or its non-parametric counterpart, the Mann-Whitney U test, to a multiple group situation increases the possibility of incorrectly rejecting the null hypothesis.

**Figure 2:**
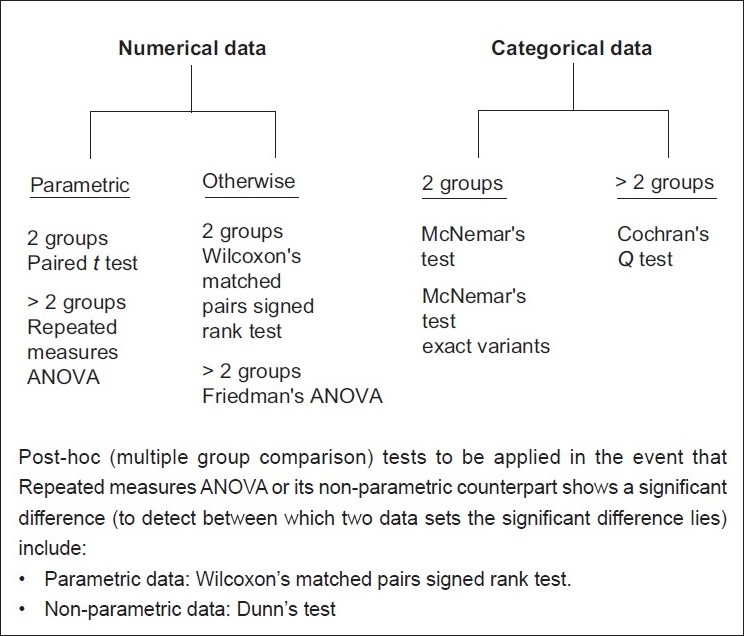
Tests to address the question: Is there a difference between groups – paired situation?

 **Question 2:** Is there a difference between groups which are paired? Pairing signifies that data sets are derived by repeated measurements (e.g. before-after measurements or multiple measurements across time) on the same set of subjects. Pairing will also occur if subject groups are different but values in one group are in some way linked or related to values in the other group (e.g. twin studies, sibling studies, parent-offspring studies). A crossover study design also calls for the application of paired group tests for comparing the effects of different interventions on the same subjects. Sometimes subjects are deliberately paired to match baseline characteristics such as age, sex, severity or duration of disease. A scheme similar to Fig. 1is followed in paired data set testing, as outlined in Fig. 2. Once again, multiple data set comparison should be done through appropriate multiple group tests followed by post hoc tests.

**Figure 3:**
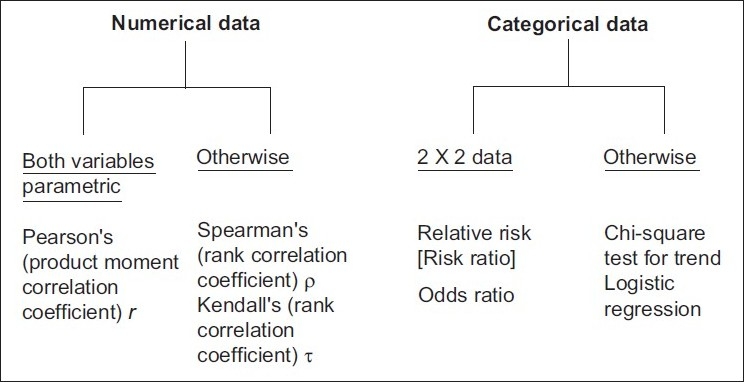
Tests to address the question: Is there an association between variables?

 **Question 3:** Is there any association between variables? The various tests applicable are outlined in Fig. 3. It should be noted that the tests meant for numerical data are for testing the association between two variables. These are correlation tests and they express the strength of the association as a correlation coefficient. An inverse correlation between two variables is depicted by a minus sign. All correlation coefficients vary in magnitude from 0 (no correlation at all) to 1 (perfect correlation). A perfect correlation may indicate but does not necessarily mean causality. When two numerical variables are linearly related to each other, a linear regression analysis can generate a mathematical equation, which can predict the dependent variable based on a given value of the independent variable.[2] Odds ratios and relative risks are the staple of epidemiologic studies and express the association between categorical data that can be summarized as a 2 × 2 contingency table. Logistic regression is actually a multivariate analysis method that expresses the strength of the association between a binary dependent variable and two or more independent variables as adjusted odds ratios.

**Figure 4:**
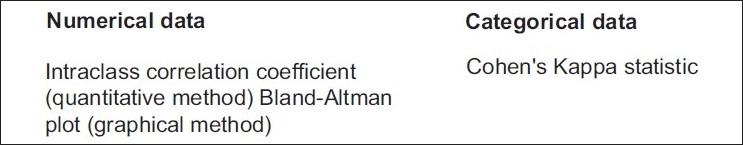
Tests to address the question: Is there an agreement between assessment (screening / rating / diagnostic) techniques?

 **Question 4:** Is there agreement between data sets? This can be a comparison between a new screening technique against the standard test, new diagnostic test against the available gold standard or agreement between the ratings or scores given by different observers. As seen from Fig. 4, agreement between numerical variables may be expressed quantitatively by the intraclass correlation coefficient or graphically by constructing a Bland-Altman plot in which the difference between two variables *x* and *y* is plotted against the mean of *x* and *y*. In case of categorical data, the Cohen’s Kappa statistic is frequently used, with kappa (which varies from 0 for no agreement at all to 1 for perfect agreement) indicating strong agreement when it is > 0.7. It is inappropriate to infer agreement by showing that there is no statistically significant difference between means or by calculating a correlation coefficient.

 **Question 5:** Is there a difference between time-to-event trends or survival plots? This question is specific to survival analysis[3](the endpoint for such analysis could be death or any event that can occur after a period of time) which is characterized by censoring of data, meaning that a sizeable proportion of the original study subjects may not reach the endpoint in question by the time the study ends. Data sets for survival trends are always considered to be non-parametric. If there are two groups then the applicable tests are Cox-Mantel test, Gehan’s (generalized Wilcoxon) test or log-rank test. In case of more than two groups Peto and Peto’s test or log-rank test can be applied to look for significant difference between time-to-event trends.

It can be appreciated from the above outline that distinguishing between parametric and non-parametric data is important. Tests of normality (e.g. Kolmogorov-Smirnov test or Shapiro-Wilk goodness of fit test) may be applied rather than making assumptions. Some of the other prerequisites of parametric tests are that samples have the same variance i.e. drawn from the same population, observations within a group are independent and that the samples have been drawn randomly from the population.

A one-tailed test calculates the possibility of deviation from the null hypothesis in a specific direction, whereas a two-tailed test calculates the possibility of deviation from the null hypothesis in either direction. When Intervention A is compared with Intervention B in a clinical trail, the null hypothesis assumes there is no difference between the two interventions. Deviation from this hypothesis can occur in favor of either intervention in a two-tailed test but in a one-tailed test it is presumed that only one intervention can show superiority over the other. Although for a given data set, a one-tailed test will return a smaller *p* value than a two-tailed test, the latter is usually preferred unless there is a watertight case for one-tailed testing.

It is obvious that we cannot refer to all statistical tests in one editorial. However, the schemes outlined will cover the hypothesis testing demands of the majority of observational as well as interventional studies. Finally one must remember that, there is no substitute to actually working hands-on with dummy or real data sets, and to seek the advice of a statistician, in order to learn the nuances of statistical hypothesis testing.
